# Two new species of *Resupinatus* (*Resupinataceae*, *Agaricales*) from China

**DOI:** 10.3897/mycokeys.140.202233

**Published:** 2026-09-18

**Authors:** Guanyu Qiu, Yupeng Ge, Hui Zeng, Tian Wang, Qin Na, Yaping Hu

**Affiliations:** 1 School of Horticulture, Ludong University, Yantai 264025, China School of Horticulture, Ludong University Yantai China; 2 Institute of Edible Mushroom, Fujian Academy of Agricultural Sciences; National and Local Joint Engineering Research Center for Breeding & Cultivation of Features Edible Fungi, Fuzhou 350014, China Institute of Edible Mushroom, Fujian Academy of Agricultural Sciences; National and Local Joint Engineering Research Center for Breeding & Cultivation of Features Edible Fungi Fuzhou China; 3 Nanjing Institute of Environmental Sciences, Ministry of Ecology and Environment, State Environmental Protection Scientific Observation and Research Station for Ecological Environment of Wuyi Mountains, Nanjing 210042, China Nanjing Institute of Environmental Sciences, Ministry of Ecology and Environment, State Environmental Protection Scientific Observation and Research Station for Ecological Environment of Wuyi Mountains Nanjing China

**Keywords:** *
Basidiomycota
*, new taxon, systematics, taxonomy

## Abstract

Within the typical saprotrophic genus *Resupinatus*, only 47 species are currently recognized worldwide. Most of these species are reported from Europe and North America, whereas only 12 species have been recorded from China. Species of *Resupinatus* are characterized by laterally or dorsally attached basidiomata, sessile or with a lateral pseudostipe, a gelatinous pileus, smooth and inamyloid basidiospores, cylindrical to coralloid cheilocystidia with apical protrusions, and pileipellis hyphae with branches or protrusions. They usually occur on dead branches and well-decayed wood in mixed coniferous and broad-leaved forests. In this study, two new species, *R. meihui***sp. nov**. and *R. cinereofuscus***sp. nov**., are recognized from China. Both species are laterally or dorsally attached to the substrate, sessile, and have lamellate hymenophores, pileus surfaces covered with tomentum or pruina, and cheilocystidia with digitate protrusions. However, *R. meihui* is distinguished by the absence of coarse felt at the point of attachment, the presence of shortly branched lamellae, and coralloid protrusions on the terminal hyphae of the pileipellis, whereas coarse felt at the point of attachment, unbranched lamellae, and smooth, thick-walled terminal hyphae in the pileipellis are observed in *R. cinereofuscus*. Morphological descriptions, habitat photographs, micrographs, and line drawings are provided for the two species, and their differences from related taxa are discussed. Phylogenetic trees inferred from ITS and nrLSU sequences using Bayesian inference and maximum likelihood analyses show that both species form independent lineages with strong support. A key to the 49 known species of *Resupinatus* is also provided.

## Introduction

The genus *Resupinatus* Nees ex Gray, which belongs to *Resupinataceae*, *Agaricales*, is mainly characterized by basidiomata that are laterally or dorsally attached, dark brown to dark gray, occasionally pale, and sessile or with a lateral pseudostipe; a gelatinous pileus; a lamellate, poroid, merulioid, or cyphelloid hymenophore; smooth and inamyloid basidiospores; cylindrical to coralloid cheilocystidia with digitate protrusions; gelatinous trama; and pileipellis hyphae with branches or protrusions (Gray 1821; Singer 1949, 1962, 1986; Thorn et al. 2005; McDonald 2015; Consiglio and Setti 2018; Vizzini et al. 2024). Species of *Resupinatus* are typical saprotrophs, usually occurring on well-decayed wood of gymnosperms or dicotyledonous trees and occasionally on gymnosperm cones, standing dead trees, or strongly decayed bamboo (Singer 1962, 1975, 1986; Thorn and Barron 1986; Thorn et al. 2005; McDonald 2015). In total, 47 species representing 92 names have been recorded for *Resupinatus* in Index Fungorum (accessed on 3 June 2026). Europe has the highest number of species, with 20, followed by North America, with 19, whereas none have been recorded from Antarctica (Li and Bau 2014; McDonald 2015; Consiglio and Setti 2018; Bijeesh et al. 2020; Akçay 2021; Yang et al. 2023; Carpouron et al. 2024; Liu et al. 2024; Vizzini et al. 2024; Dong et al. 2025; Triantafyllou et al. 2026; Zhuang et al. 2026). To date, 12 species of *Resupinatus*, including eight newly described species, have been recorded from China (Yang et al. 2023; Liu et al. 2024; Dong et al. 2025; Zhuang et al. 2026). The highest number of species has been reported from Northeast China, with seven species, followed by Southwest China, with five species (Teng 1963; Huang 1998; Lu 2000; Bau et al. 2007, 2013; Wu et al. 2009, 2011; Li and Bau 2014; Yang et al. 2023; Liu et al. 2024; Dong et al. 2025; Zhuang et al. 2026).

Because differing delimitation criteria have been applied to the genus *Resupinatus* by various authors, its taxonomic status has long been controversial and was not settled until recently (Gray 1821; Fries 1874; Kühner 1926, 1980; Pilát 1935; Jülich 1981; Singer 1986). The genus *Resupinatus* was first proposed by Gray within *Hymenotheceae*, *Agaricales*, characterized by dorsally attached basidiomata, an orbicular and membranaceous pileus, and the absence of a stipe, annulus, and volva (Gray 1821). However, micromorphological characters were not provided, and only the type species *R. applicatus* (Batsch) Gray was recorded (Gray 1821). Subsequently, based on both macromorphological and micromorphological features, *Resupinatus* was placed in the tribe *Resupinateae*, *Tricholomataceae*, by Singer (Singer 1948, 1949, 1962, 1986), who also revised the concept as follows: pleurotoid basidiomata that are dorsally adnate or with a pseudostipe, a gelatinous pileus, a lamellate hymenophore, smooth and inamyloid basidiospores, dendroid cheilocystidia, and a pileipellis composed of hyphae with stellate protrusions or exhibiting a Rameales structure (Singer 1948, 1949, 1962, 1986). In 2015, phylogenetic relationships were reconstructed by McDonald based on ITS and nrLSU sequences, indicating that species of the tribe *Resupinateae* formed a strongly supported independent lineage within *Agaricales* (McDonald 2015). The lineages of *Rhodocyphella* W.B. Cooke, *Stromatocyphella* W.B. Cooke, and *Stigmatolemma* Kalchbr. were included within the *Resupinatus* clade, so the species of these three genera were transferred into *Resupinatus* (McDonald 2015). The monophyly of *Resupinatus* was further supported by a large-scale multigene phylogenetic study of *Agaricomycetes* conducted by Vizzini et al. (2024), and the tribe *Resupinateae* was subsequently elevated to family rank as *Resupinataceae* (Jülich 1981; Vizzini et al. 2024).

At this point, the taxonomic status of *Resupinatus* has been clarified. However, for a long time, there has been a lack of type specimens from China in most molecular systematic studies of *Resupinatus*. Moreover, there is a significant gap between the number of recorded *Resupinatus* species in China and the worldwide total. At present, only 12 species have been recorded in China, whereas 47 species have been recorded globally. During field collection, nearly 40 specimens were obtained. This indicates that *Resupinatus* is widely distributed in China. Research on *Resupinatus* in China needs to be accelerated to provide Chinese samples for further exploration of the origin and evolution of the genus.

## Materials and methods

### Specimen collection and morphological study

The specimens examined in this study were collected from the Guangxi Zhuang Autonomous Region and Hubei, Shandong, Yunnan, and Zhejiang provinces, China. High-resolution photographs of fresh basidiomata, attached substrates, and habitats were taken using an Olympus E-M1 Mark III camera equipped with M. Zuiko Digital ED 12–40 mm and 60 mm lenses (Olympus, Tokyo, Japan). Each collection was assigned a field number as its voucher number. The size and color of basidiomata, pileus surface texture, striation, tomentum, lamella morphology and number (*L* = number of lamellae, *l* = number of lamellulae), presence or absence of coarse felt at the point of attachment, substrate type, habitat, and forest type were recorded in detail. Color descriptions followed Ridgway (1912). Specimens were dried in a portable dryer at 40 °C (A. & J. Stöckli AG, Netstal, Switzerland) for approximately 48 h (Hu et al. 2022). Fully dried specimens were stored in sealed bags with color-indicating silica gel. A portion of the lamellae from the basidiomata was taken for DNA extraction and molecular analyses, and the specimens were deposited in the Fungarium of the Fujian Academy of Agricultural Sciences (**FFAAS**), China.

Micromorphological characters were examined using a Lab A1 microscope (Carl Zeiss AG, Jena, Germany). Dried specimens were rehydrated in a 5% KOH solution, stained with a 1% Congo red solution when necessary, and tested in Melzer’s reagent to assess amyloid reactions of basidiospores and other tissues (Vizzini et al. 2024). Microscopic structures were photographed and measured using ZEN 2.3 (blue edition) software (Carl Zeiss Microscopy GmbH, Jena, Germany). For each basidioma, 25 mature basidiospores in side view were randomly selected and measured. Basidiospore measurements are presented as follows: [a/b/c] (d)e–***f***–g(h) × (i)j–***k***–l(m) µm, [Q = (n)o–p(q), Qm = ***r*** ± s], where a, b, and c indicate the number of basidiospores, basidiomata, and specimens measured, respectively; d and h represent the extreme minimum and maximum values of basidiospore length, each accounting for 5% of the measurements; e and g represent the minimum and maximum values within the 90% range; and f represents the mean value; basidiospore width values (i–m) are given in the same manner (Bandala and Montoya 2000; Dramani et al. 2020; Na et al. 2021). Q indicates the length/width ratio of a basidiospore, and Qm represents the mean Q value of all measured basidiospores ± sample standard deviation (Bandala and Montoya 2000; Dramani et al. 2020; Na et al. 2021). At least 20 randomly selected structures were measured for other microscopic characters. The shape, protrusions, and contents of basidia, cheilocystidia, and pleurocystidia (when present) were also recorded, together with the structure of the trama and pileipellis and the presence or absence of thick walls, branches, and protrusions in the hyphae (McDonald 2015; Consiglio and Setti 2018).

Habitat photographs, micrographs, field notes, and line drawings were used to prepare habitat and microscopic plates. Line drawings were scanned in TIFF format using a Canon LiDE 120 scanner (Canon, Tokyo, Japan), and the plates were edited and arranged in Adobe Photoshop CC 2024.

### DNA extraction and PCR amplification

Genomic DNA was extracted from dried specimens using the NuClean Plant Genomic DNA Kit (Beijing ComWin Biotech Co., Ltd., Beijing, China). The primer pairs ITS1/ITS4 and LR0R/LR7 were used to amplify the internal transcribed spacer (ITS) region and the large subunit nuclear ribosomal RNA (nrLSU) region, respectively (White et al. 1990; Hopple Jr and Vilgalys 1999; McDonald 2015; Consiglio and Setti 2018). PCR amplification was performed in a 25 μL reaction mixture containing 12.5 μL of 2 × Utaq PCR MasterMix (Beijing Zoman Biotechnology Co., Ltd., Beijing, China), 8.5 μL of ddH_2_O, 1 μL of each primer, and 2 μL of DNA template. The amplification conditions for ITS were as follows: initial denaturation at 94 °C for 4 min; 34 cycles of denaturation at 94 °C for 45 s, annealing at 52 °C for 45 s, and extension at 72 °C for 1 min; followed by a final extension at 72 °C for 10 min (White et al. 1990). The amplification conditions for nrLSU were as follows: initial denaturation at 95 °C for 5 min; 30 cycles of denaturation at 95 °C for 30 s, annealing at 55 °C for 30 s, and extension at 72 °C for 1 min; followed by a final extension at 72 °C for 10 min (Hopple Jr and Vilgalys 1999). PCR products were sent to Beijing BGI-Liuhe Biotech Co., Ltd. (Beijing, China) for sequencing.

### Phylogenetic analyses

Sequence quality was checked using BioEdit version 7.2.5 (Hall 1999). Sequences with overlapping peaks were subjected to plasmid ligation and transformation to obtain high-quality sequences. Phylogenetic analyses were conducted using Bayesian inference (BI) and maximum likelihood (ML). Reference sequences were selected based on BLAST searches (https://blast.ncbi.nlm.nih.gov/) and previous phylogenetic studies, with priority given to type or representative sequences of accepted *Resupinatus* species when available (McDonald 2015; Consiglio and Setti 2018). *Pleurotus ostreatus* (Jacq.) P. Kumm. was selected as the outgroup (McDonald 2015; Consiglio and Setti 2018). The sequence matrix was aligned using MAFFT version 7 with the auto strategy, which included FFT-NS-1, FFT-NS-2, FFT-NS-i, or L-INS-i as appropriate (Katoh et al. 2002; Tamura et al. 2011). Missing data were coded as gaps, and multi-copy sites were represented by degenerate bases. The alignment was manually adjusted in BioEdit version 7.2.5 (Hall 1999). Sequences were concatenated in MEGA 7.0.26, and missing regions were filled with gaps.

For BI, the best-fit model for each gene region was selected using MrModeltest 2.3 (Nylander 2004). In MrBayes v3.2.6, Markov chain Monte Carlo analyses were run with four chains for 10,000,000 generations, sampling every 1,000 generations. The analyses were checked to ensure that the average standard deviation of split frequencies was below 0.01. The first 25% of sampled trees were discarded as burn-in, and the results were summarized using the “sump burnin” and “sumt burnin” commands (Ronquist and Huelsenbeck 2003). Tracer v1.7.2 was used to assess the effective sample size (ESS > 200) and the average potential scale reduction factor (1.0 < PSRF < 1.1), confirming that the BI analysis had reached stationarity (Rambaut et al. 2018). For ML analysis, the best-fit model for each partition was selected in raxmlGUI 2.0, and 1,000 rapid bootstrap (BS) replicates were performed (Edler et al. 2021). Phylogenetic trees were visualized in FigTree v1.4.3 and edited in Adobe Illustrator 2020.

## Results

### Phylogenetic analyses

**Figure 1.**
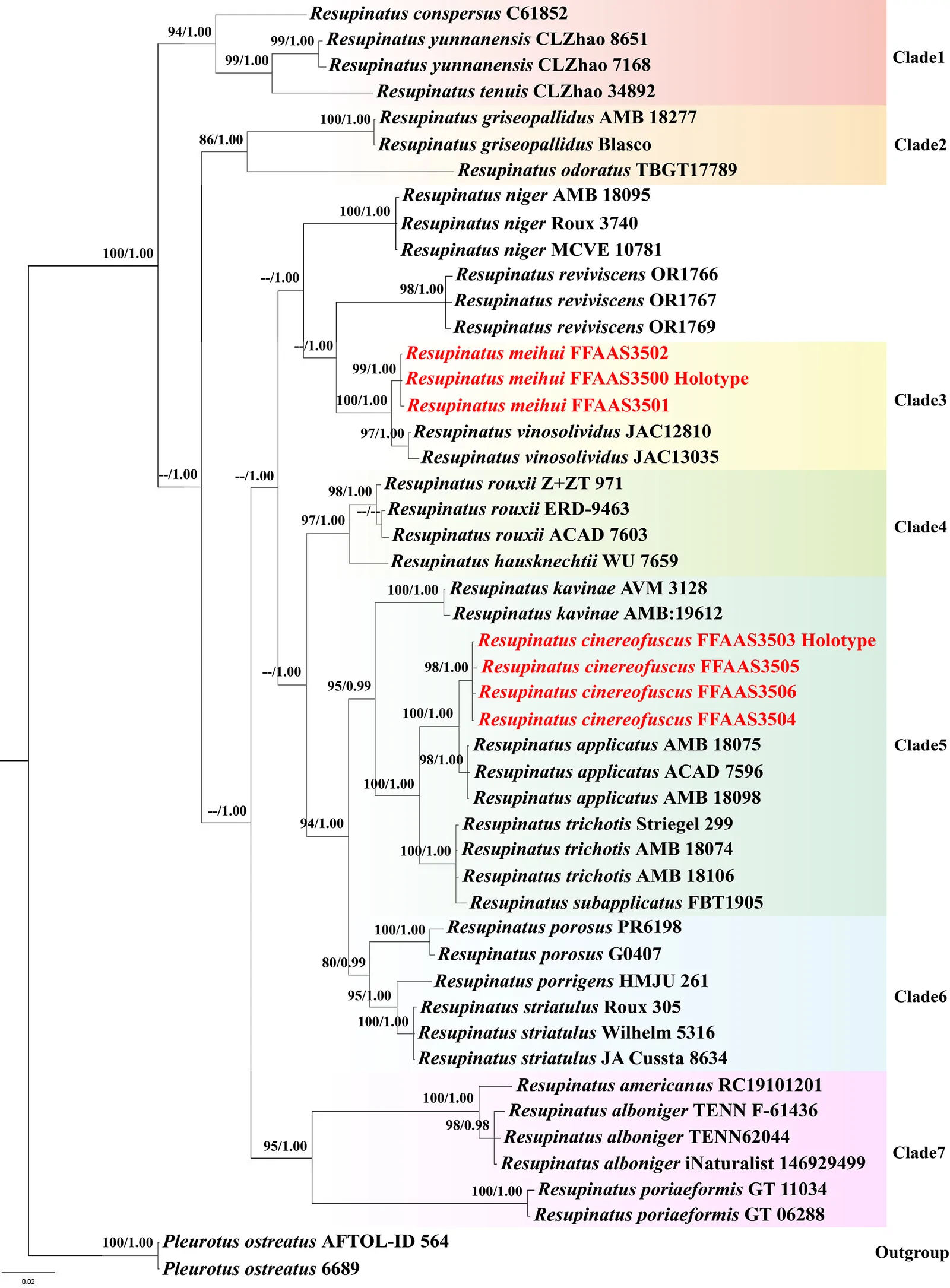
Phylogenetic tree inferred from Bayesian inference (BI) analysis based on the ITS + nrLSU dataset. Branches with maximum likelihood bootstrap values greater than 75% and Bayesian posterior probability values greater than 0.95 are indicated. Different clades are marked with different colors. The new species are indicated in red.

The sequence matrix included 79 sequences from 23 species, with 47 ITS and 32 nrLSU sequences. Of these, 65 sequences were downloaded from GenBank, including 40 ITS and 25 nrLSU sequences, and 14 sequences were newly generated in this study. Detailed information is provided in Table 1. The combined dataset comprised 1,555 nucleotide positions, including 274 bp of ITS1, 173 bp of 5.8S, 232 bp of ITS2, and 876 bp of nrLSU. In the Bayesian inference analysis, the best-fit models for ITS1, 5.8S, and ITS2 were GTR+G, HKY+I, and GTR+I+G, respectively; the best-fit model for nrLSU was GTR+I+G. After 10,000,000 generations of Markov chain Monte Carlo (MCMC) analyses, the average standard deviation of split frequencies was 0.002437, the effective sample size (ESS) was 7405.6, and the average potential scale reduction factor (PSRF) was 1.000. In the maximum likelihood analysis, the best-fit models for ITS1, 5.8S, and ITS2 were TPM2uf+G, JC, and TPM2uf+G, respectively; the best-fit model for nrLSU was GTR+I+G. The maximum likelihood analysis was conducted with 1,000 nonparametric bootstrap replicates, yielding a final log-likelihood value of −7607.472531. The Bayesian inference and maximum likelihood analyses yielded similar topologies; therefore, the Bayesian tree is shown in Fig. 1. Maximum likelihood bootstrap values (BS) greater than 75% and Bayesian posterior probabilities (BPP) greater than 0.95 are indicated at the nodes.

**Table 1.** Specimens of *Resupinatus* used in phylogenetic analyses, with geographic origins and GenBank accession numbers.

**Species**	**Voucher/strain no**.	**Locality**	**GenBank no**.		**Reference**
			**ITS**	**nrLSU**	
*R. alboniger*	TENN F-61436	America	KP026236	—	Petersen et al. 2015
*R. alboniger*	TENN62044	America	FJ596893	—	Hughes et al. 2009
*R. alboniger*	iNaturalist 146929499	America	OR081334	—	Unpublished
*R. americanus*	RC19101201	France	OM397444	—	Unpublished
*R. applicatus*	AMB 18075	Italy	KU355368	KU355411	Consiglio and Setti 2018
*R. applicatus*	AMB 18098	Spain	MH137821	MH430596	Consiglio and Setti 2018
*R. applicatus*	ACAD 7596	Italy	KU355367	—	Consiglio and Setti 2018
***R. cinereofuscus***	**FFAAS3503 Holotype**	**China**	**PZ456431**	**PZ456470**	**This study**
***R. cinereofuscus***	**FFAAS3504**	**China**	**PZ456432**	**PZ456471**	**This study**
***R. cinereofuscus***	**FFAAS3505**	**China**	**PZ456433**	**PZ456472**	**This study**
***R. cinereofuscus***	**FFAAS3506**	**China**	**PZ456434**	**PZ456473**	**This study**
*R. conspersus*	C61852	America	AY571061	AY571024	Bodensteiner et al. 2004
*R. griseopallidus*	AMB 18277	Spain	MH137823	MH165881	Consiglio and Setti 2018
*R. griseopallidus*	Blasco	Spain	MG553642	MG553649	Consiglio and Setti 2018
*R. hausknechtii*	WU 7659	Italy	KU355370	KU355412	Consiglio and Setti 2018
*R. kavinae*	AVM 3128	Spain	MG553643	MG553650	Consiglio and Setti 2018
*R. kavinae*	AMB:19612	Spain	OR863477	OR863543	Vizzini et al. 2024
***R. meihui***	**FFAAS3500 Holotype**	**China**	**PZ456428**	**PZ456467**	**This study**
***R. meihui***	**FFAAS3501**	**China**	**PZ456429**	**PZ456468**	**This study**
***R. meihui***	**FFAAS3502**	**China**	**PZ456430**	**PZ456469**	**This study**
*R. niger*	AMB 18095	Italy	KU355371	KU355413	Consiglio and Setti 2018
*R. niger*	Roux 3740	Italy	KU355372	KU355414	Consiglio and Setti 2018
*R. niger*	MCVE 10781	Italy	KU355331	KU355395	Consiglio and Setti 2018
*R. odoratus*	TBGT17789	India	MT452498	—	Bijeesh et al. 2020
*R. poriaeformis*	GT 06288	Spain	MH137826	—	Consiglio and Setti 2018
*R. poriaeformis*	GT 11034	Spain	MH137825	—	Consiglio and Setti 2018
*R. porosus*	PR6198	Spain	—	DQ017064	Thorn et al. 2005
*R. porosus*	G0407	Hungary	—	MK278554	Varga et al. 2019
*R. porrigens*	HMJU 261	China	OP727724	OP727807	Liu et al. 2024
*R. reviviscens*	OR1766	Thailand	PQ036934	PQ036940	Carpouron et al. 2024
*R. reviviscens*	OR1767	Thailand	PQ036935	PQ036941	Carpouron et al. 2024
*R. reviviscens*	OR1769	Thailand	PQ036937	PQ036942	Carpouron et al. 2024
*R. rouxii*	Z+ZT 971	Spain	MH168326	MH190787	Consiglio and Setti 2018
*R. rouxii*	ERD-9463	Spain	OP289290	—	Unpublished
*R. rouxii*	ACAD 7603	Italy	KU355369	—	Consiglio and Setti 2018
*R. striatulus*	Roux 305	Italy	KU355373	—	Consiglio and Setti 2018
*R. striatulus*	Wilhelm 5316	Spain	MH137831	MH169342	Consiglio and Setti 2018
*R. striatulus*	JA Cussta 8634	Spain	MH137829	MH430597	Consiglio and Setti 2018
*R. subapplicatus*	FBT1905	Australia	MT856383	—	Unpublished
*R. tenuis*	CLZhao 34892	China	PV197932	PV197946	Dong et al. 2025
*R. trichotis*	AMB 18074	Italy	KU355378	KU355416	Consiglio and Setti 2018
*R. trichotis*	AMB 18106	Italy	KU355377	—	Consiglio and Setti 2018
*R. trichotis*	Striegel 299	Italy	KU355381	—	Consiglio and Setti 2018
*R. vinosolividus*	JAC12810	New Zealand	PX057404	—	Cooper 2012
*R. vinosolividus*	JAC13035	New Zealand	PX057405	—	Cooper 2012
*R. yunnanensis*	CLZhao 7168	China	OP901838	—	Yang et al. 2023
*R. yunnanensis*	CLZhao 8651	China	OP901839	OP904197	Yang et al. 2023
*P. ostreatus*	AFTOL-ID 564	America	AY854077	AY657015	Unpublished
*P. ostreatus*	6689	Czech	AY450345	—	Petersen and Krisai-Greilhuber 1996

Remarks: Newly generated sequences are emphasized in bold; “—” shows missing sequence.

The phylogenetic analyses resolved seven well-supported clades (Fig. 1). *Resupinatus meihui* and *R. cinereofuscus* were located in Clade 3 (BS/BPP = 100/1.00) and Clade 5 (BS/BPP = 95/0.99), respectively. Clade 3 comprised only two species, *R. meihui* and *R. vinosolividus* (Segedin) J.A. Cooper, each forming a highly supported, independent branch (BS/BPP = 99/1.00 for *R. meihui*, BS/BPP = 97/1.00 for *R. vinosolividus*). Five taxa, *R. applicatus*, *R. cinereofuscus*, *R. kavinae* (Pilát) M.M. Moser, *R. subapplicatus* (Cleland) Grgur., and *R. trichotis* (Pers.) Singer, clustered together with high statistical support (BS/BPP = 95/0.99) in Clade 5. Within this clade, *R. cinereofuscus* constituted a monophyletic lineage that was closely related to *R. applicatus* (BS/BPP = 100/1.00), from Italy, but distantly related to the other three species.

### Taxonomy

#### Resupinatus meihui

Taxon classificationFungiAgaricalesPleurotaceae

G.Y. Qiu, Q. Na & Y.P. Ge sp. nov.

8993015D-4B99-5646-8F56-BE403C7BA547

863895

Figs 2, 3, 4

##### Chinese name.

煤灰伏褶菌 Mei Hui Fu Zhe Jun.

**Figure 2.**
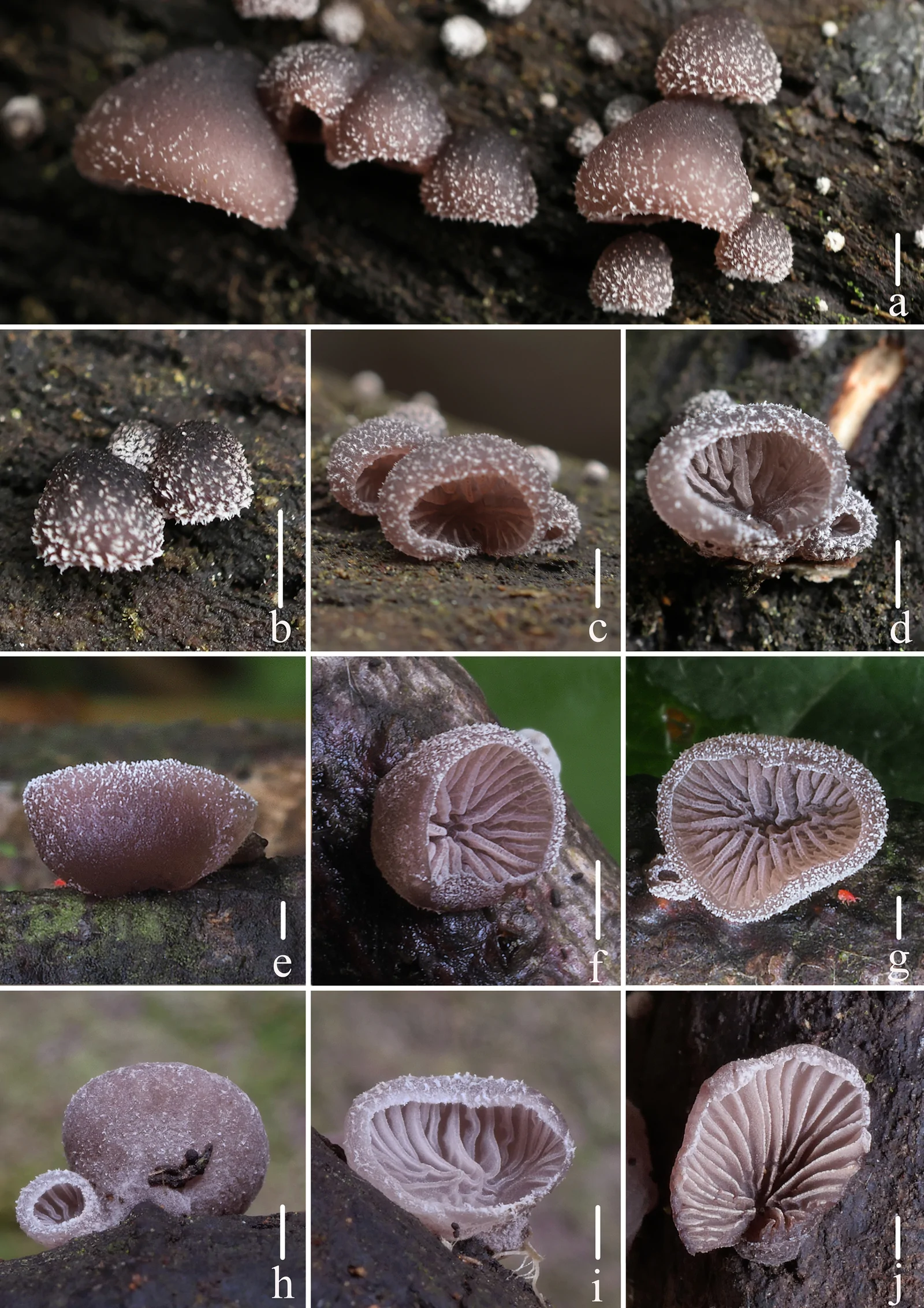
Basidiomata of *Resupinatus meihui*. **a–d**. *FFAAS3501*; **e–g**. *FFAAS3500* (holotype); **h–j**. *FFAAS3502*. Scale bars: 1 mm (**a–j**). Photos **a–d** by Paopao; **e–g** by Yupeng Ge; **h–j** by Liangliang Qi.

**Figure 3.**
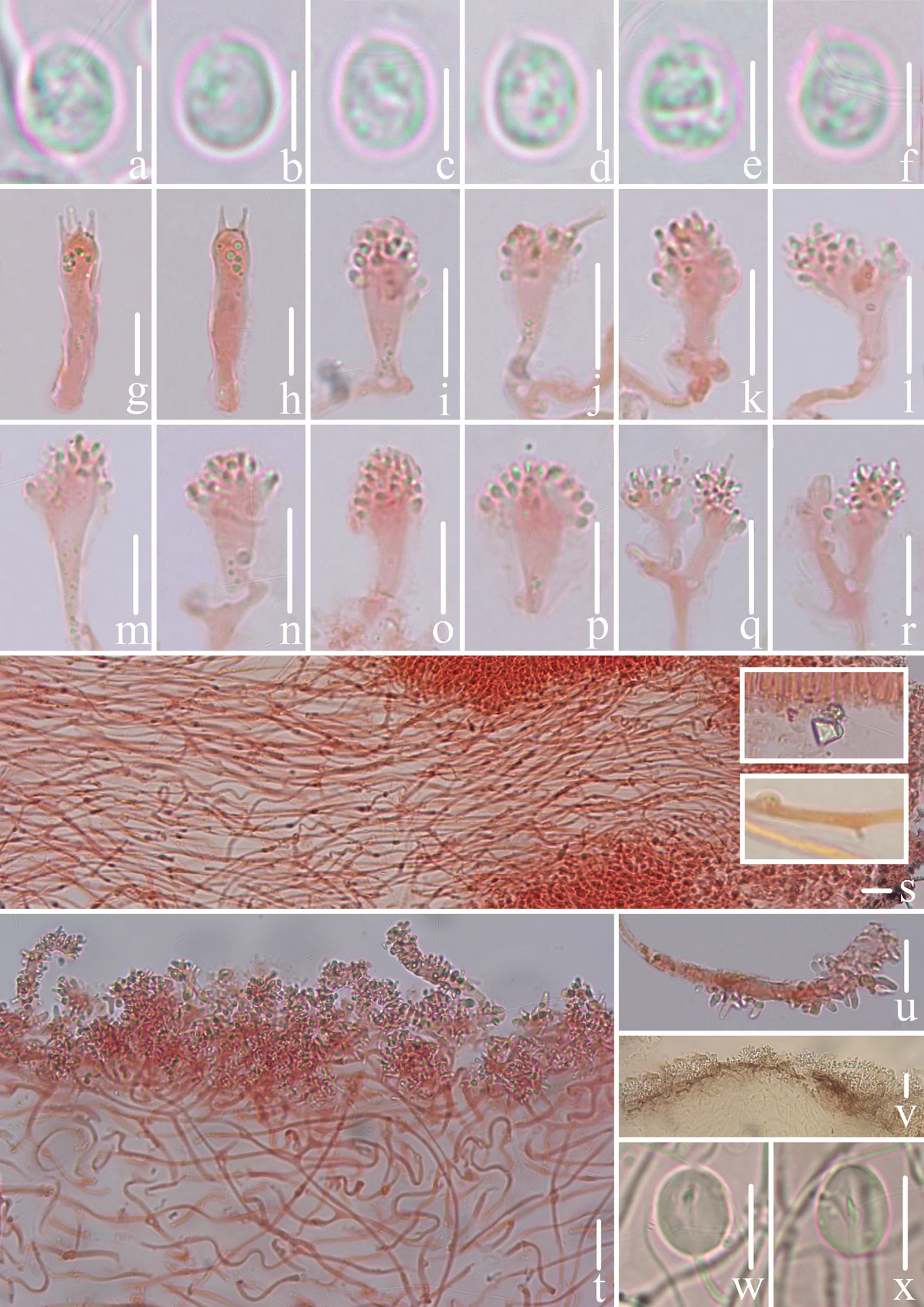
Micromorphological features of *Resupinatus meihui FFAAS3500* (holotype). **a–f**. Basidiospores; **g–h**. Basidia; **i–r**. Cheilocystidia; **s**. Lamellar trama; **t**. Pileipellis; **u**. Terminal cell of the pileipellis; **v**. Intracellular pigmentation of the pileipellis; **w–x**. Gloeocystidia of the pileipellis. Scale bars: 5 μm (**a–f**); 15 μm (**g–r, w–x**); 8 μm (**s–u**); 10 μm (**v**). Structures **a–f, v–x** were rehydrated in a 5% KOH solution, and **g–u** were stained with a 1% Congo red solution. Photos by Yupeng Ge and Guanyu Qiu.

**Figure 4.**
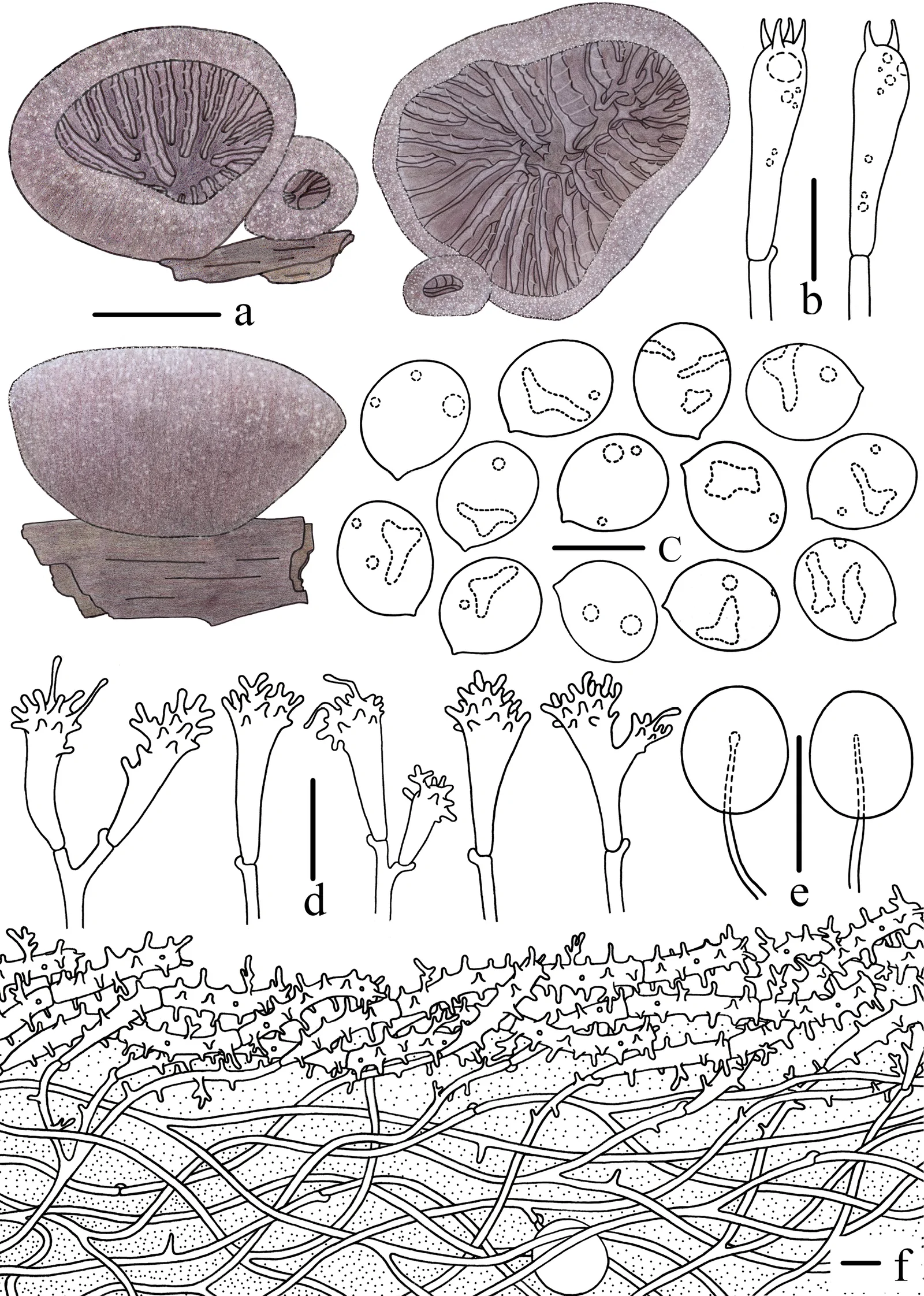
Morphological features of *Resupinatus meihui FFAAS3500* (holotype). **a**. Basidiomata; **b**. Basidia; **c**. Basidiospores; **d**. Cheilocystidia; **e**. Gloeocystidia; **f**. Pileipellis. Scale bars: 2 mm (**a**); 15 μm (**b, d, e**); 5 μm (**c, f**). Drawing by Guanyu Qiu.

##### Diagnosis.

Pileus densely tomentose, tomentum tufted, lamellae with short lateral branches, and coarse felt absent at the point of attachment to the substrate. Differs from *R. vinosolividus* in having broadly ellipsoid to subglobose basidiospores and pileipellis terminal cells densely covered with irregular digitate protrusions.

##### Holotype.

China • Hubei Province, Yichang City, Wufeng County, Houhe National Nature Reserve, 18 June 2024, Guanyu Qiu, Yupeng Ge, Qin Na, Hui Zeng, Yaping Hu, Jingwen Guo, and Lijun Wang, 1,652 m alt., *FFAAS3500* (collection no. NJ5297).

##### Etymology.

The epithet *meihui* is derived from the Chinese words “mei” and “hui”, alluding to the delicate white tufted tomentum scattered on the grayish pileus surface.

##### Description.

***Pileus*** 1.2–6.1 mm in diam., at first campanulate to ungulate, margin incurved, near attachment point Deep Neutral Gray (LIIINEUTRAL GRAYi) to Slate-Gray (LIIICARBON GRAYi), center Light Purplish Gray (LIII67’’’’’b) to Deep Purplish Gray (LIII67’’’’’i), gradually paler towards margin, Pallid Purplish Gray (LIII67’’’’’f) to Pale Vinaceous-Drab (XLV5’’’’d); when mature, slightly expanded, cupulate to bowl-shaped, near attachment point Vinaceous-Slate (L69’’’’b) to Deep Vinaceous-Gray (L69’’’’i), center Heliotrope Gray (L65’’’’d) to Vinaceous Gray (L69’’’’d), margin Light Heliotrope Gray (L65’’’’f) to Light Vinaceous Gray (L69’’’’f); surface densely covered with white tufted tomentum, becoming sparser towards attachment point, tomentum persistent after drying; not striate, not hygrophanous. ***Context*** up to 2 mm thick, translucent, gelatinous, tough, elastic, not fragile. ***Hymenophore*** lamellate; ***lamellae*** < 1 mm broad, *L* = 5–9, *l* = 1–7, free, Pale Vinaceous-Drab (XLV5’’’’d) to Pale Brownish Drab (XLV9’’’’d) when young, Light Plumbago Gray (L61’’’’f) to Light Vinaceous-Gray (L69’’’’f) when mature, surface pruinose, gelatinous, with short lateral branches. ***Stipe*** absent, basidiomata laterally or dorsally attached, coarse felt absent at the point of attachment to the substrate. Odour and taste indistinct.

***Basidiospores*** (150/6/3) 5.0–***5.4***–5.9 × 4.0–***4.3***–4.8 μm, [Q = (1.08)1.13–1.35, Qm = ***1.25*** ± 0.01] [holotype (50/2/1) 5.0–***5.4***–5.8 × 4.0–***4.3***–4.7 μm, Q = (1.15)1.19–1.32, Qm = ***1.25*** ± 0.01], subglobose to broadly ellipsoid, smooth, thin-walled, hyaline, with circular or irregular guttules, inamyloid. ***Basidia*** 20–40 × 5–9 μm, cylindrical to clavate, 2- or 4-spored, sterigmata 2.8–5.5 μm long, thin-walled, smooth, hyaline, with guttules. ***Cheilocystidia*** cylindrical to clavate, 10–25 × 3–16 μm, constricted at base, apically enlarged, densely covered with irregular verrucose or digitate protrusions 3.0–8.0 μm long, thin-walled, hyaline. ***Pleurocystidia*** absent. ***Pileipellis*** a trichocutis, with a gelatinous layer, non-dextrinoid, composed of cylindrical hyphae 2–3 μm in diam., thin-walled, hyaline, occasionally with spinose protrusions 4.3–5.2 μm long; terminal cells 13–44 × 4–5 μm, with dense, irregular digitate protrusions 3.1–8.9 μm long, containing brown intracellular pigment; terminal hyphae interwoven, forming trichoderm; gloeocystidia occasionally present, subglobose to ellipsoid, 9–17 × 7–15 μm. ***Lamellar trama*** subregular, with a gelatinous layer, non-dextrinoid, composed of cylindrical hyphae 1–3 μm in diam., interwoven to subparallel, with spinose protrusions 4.1–4.9 μm long, thin-walled, hyaline, crystals occasionally present. Clamp connections present in all tissues.

##### Habit and habitat.

Gregarious on well-decayed wood of broad-leaved trees in summer and autumn, particularly on species of *Castanopsis*, *Lithocarpus*, *Phoebe*, and *Cinnamomum*.

##### Known distribution.

Guangxi Zhuang Autonomous Region, Hubei Province, and Yunnan Province, China.

##### Additional specimens examined.

China • Yunnan Province, Chuxiong Yi Autonomous Prefecture, Lufeng County, Tuo’an Township, Douzui, 15 June 2023, Guanyu Qiu, Paopao, and Meihui, 2,355 m alt., *FFAAS3501* (collection no. GN2761); • Guangxi Zhuang Autonomous Region, Guigang City, Pingnan County, Mengshan Shiyading, 18 February 2025, Guanyu Qiu, Liangliang Qi, and Yongqiang Hu, 1,055 m alt., *FFAAS3502* (collection no. GN2773).

##### Notes.

*Resupinatus meihui* resembles *R. vinosolividus* and *R. cinerascens* (Cleland) Grgur., which were originally described from New Zealand and Australia, respectively (Segedin 1993; Cooper 2012; McDonald 2015). The three species share the same gelatinous basidiomata, tomentose pileus, lamellate hymenophore, and branched lamellae (Segedin 1993; Cooper 2012; McDonald 2015). However, *R. vinosolividus* has ellipsoid to oblong or sometimes slightly ovoid basidiospores, as well as pileipellis hyphae with branched or stellate protrusions (Segedin 1993; Cooper 2012). *Resupinatus cinerascens* is characterized by a larger pileus, up to 15 mm in diam., ellipsoid to ovoid basidiospores, and a gregarious growth habit on decayed wood of *Eucalyptus* and other conifers (McDonald 2015). *Resupinatus merulioides* Redhead & Nagas. is also similar to *R. meihui* in morphology, but the former can be distinguished by the hoary white coating on the pileus surface when dry, lamellar sides interconnecting and becoming nearly poroid in appearance, and pileipellis hyphae with tapered to tibiiform protrusions (Redhead and Nagasawa 1987). In addition, *R. applicatus* could be confused with *R. meihui*, but it differs in having mature basidiomata with a spatulate to flabelliform shape, unbranched lamellae, and a gray to brown coarse felt at the attachment point (Consiglio and Setti 2018).

#### Resupinatus cinereofuscus

Taxon classificationFungiAgaricalesPleurotaceae

G.Y. Qiu, Q. Na & Y.P. Ge sp. nov.

A8C67CF4-F395-5B41-A6F5-05E94B4A139A

863896

Figs 5, 6, 7

##### Chinese name.

灰褐伏褶菌 Hui He Fu Zhe Jun.

**Figure 5.**
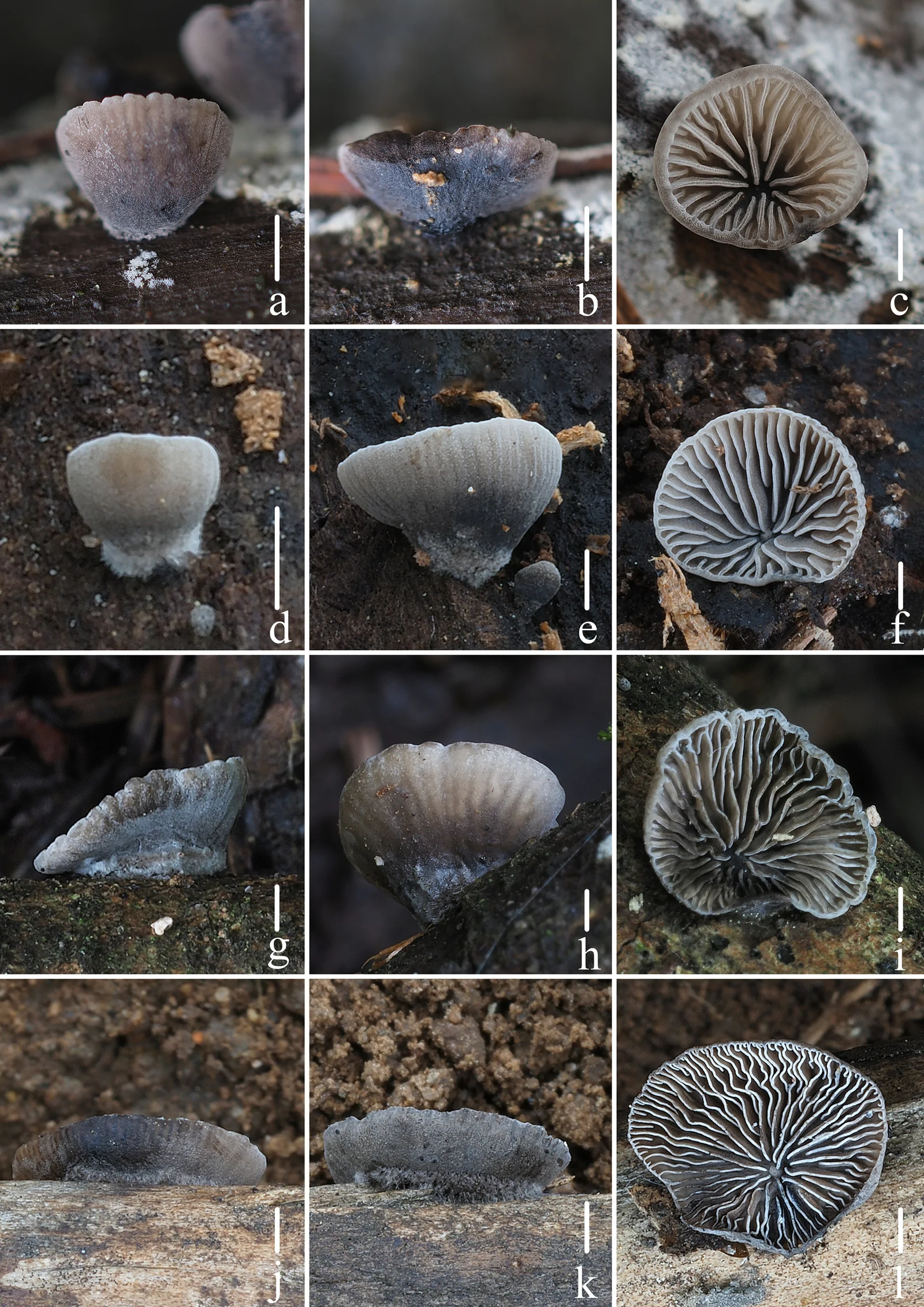
Basidiomata of *Resupinatus cinereofuscus*. **a–c**. *FFAAS3504*; **d–f**. *FFAAS3505*; **g–i**. *FFAAS3506*; **j–l**. *FFAAS3503* (holotype). Scale bars: 1 mm (**a–l**). Photos by Yupeng Ge.

**Figure 6.**
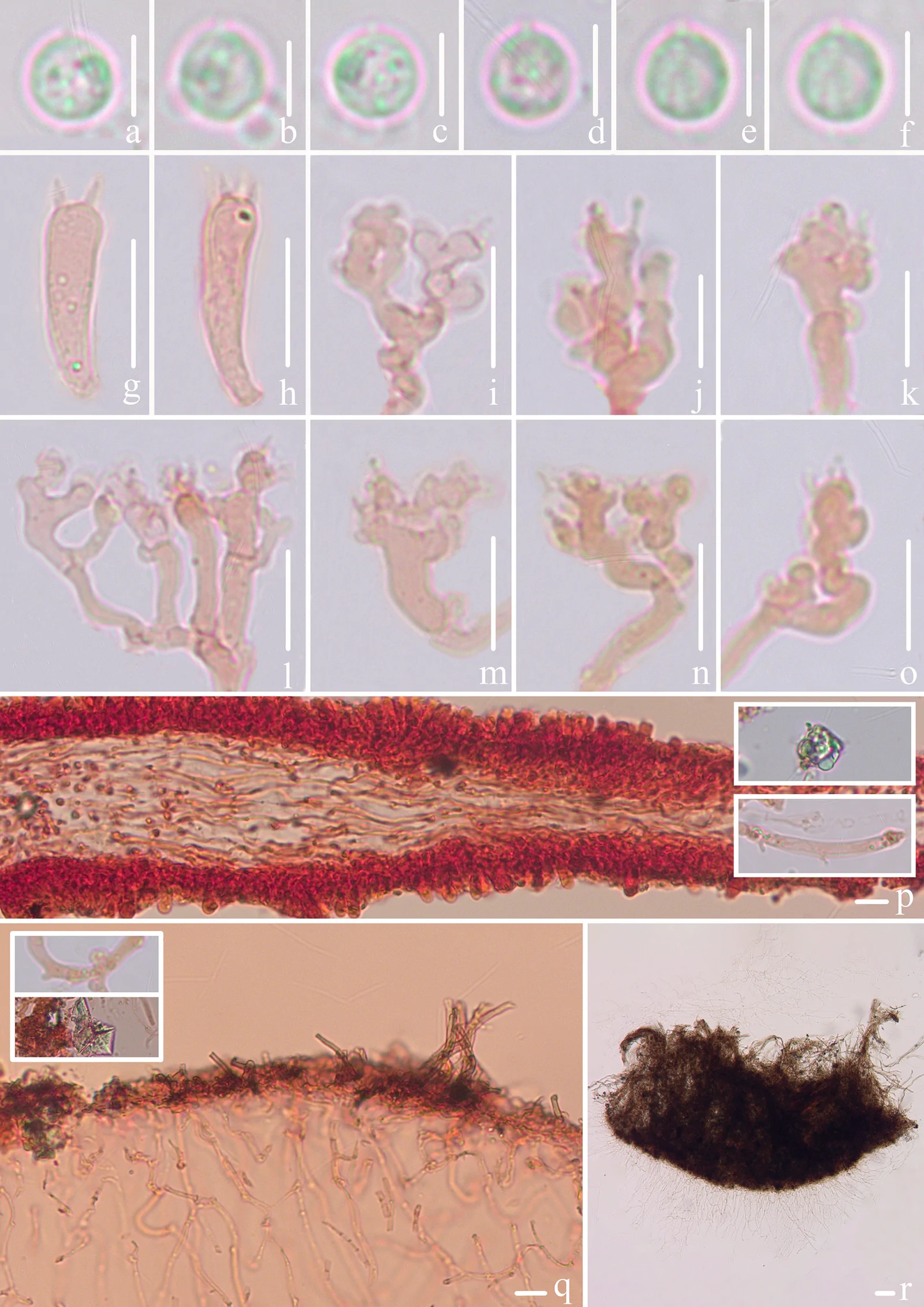
Micromorphological features of *Resupinatus cinereofuscus FFAAS3503* (holotype). **a–f**. Basidiospores; **g, h**. Basidia; **i–o**. Cheilocystidia; **p**. Lamellar trama; **q, r**. Pileipellis (**q**. Thinly sliced; **r**. Thickly sliced). Scale bars: 4 μm (**a–f**); 10 μm (**g–o, r**); 8 μm (p, q). Structures **a–f, r** were rehydrated in a 5% KOH solution, and **g–q** were stained with a 1% Congo red solution. Photos by Yupeng Ge and Guanyu Qiu.

**Figure 7.**
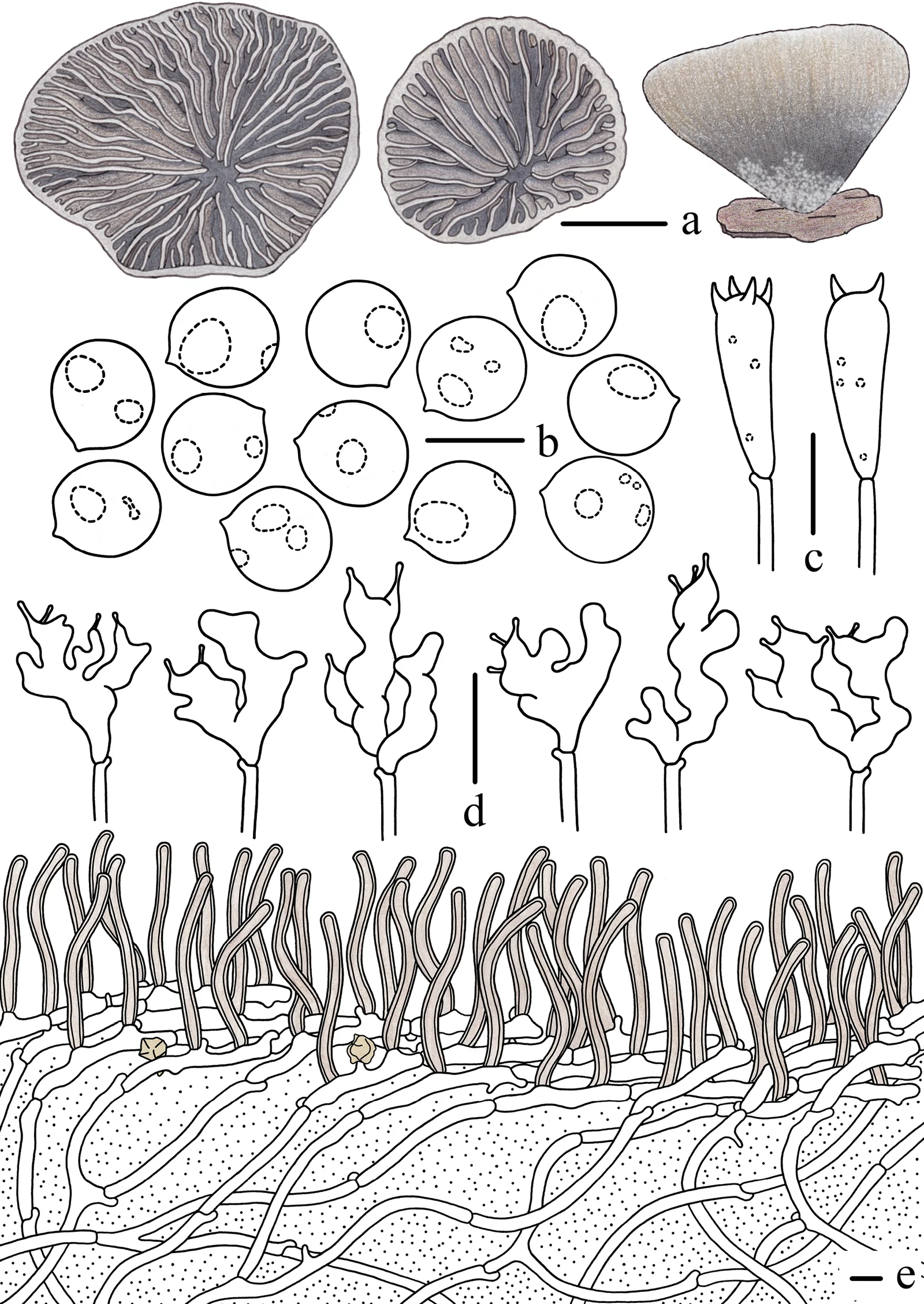
Morphological features of *Resupinatus cinereofuscus FFAAS3503* (holotype). **a**. Basidiomata; **b**. Basidiospores; **c**. Basidia; **d**. Cheilocystidia; **e**. Pileipellis. Scale bars: 2 mm (**a**); 4 μm (**b, e**); 10 μm (**c, d**). Drawing by Guanyu Qiu.

##### Diagnosis.

Pileus faintly striate, pruinose, with coarse felt at the point of attachment to the substrate. Differs from *R. applicatus* by the faintly striate pileus, the absence of pleurocystidia, and branched terminal pileipellis hyphae that give rise to erect, smooth, and thick-walled hyphae.

##### Holotype.

China • Shandong Province, Tai’an City, Daiyue District, Culai Mountain National Forest Park, 23 July 2021, Guanyu Qiu, Yupeng Ge, Qin Na, Hui Zeng, Zewei Liu, Yulan Sun, and Menghui Han, 883 m alt., *FFAAS3503* (collection no. GN1286).

##### Etymology.

The epithet *cinereofuscus* refers to the grayish-brown color of the basidiomata.

##### Description.

***Pileus*** 2.4–12.1 mm in diam., at first shell-shaped to ungulate, margin slightly incurved, undulate, near attachment point Hathi Gray (LII35’’’’’b) to *Plumbeous (LII49’’’’’b), center Pale Quaker Drab (LI1’’’’’d) to Pale Mouse Gray (LI15’’’’’d), gradually paler towards margin, Pallid Quaker Drab (LI1’’’’’f) to Pallid Mouse Gray (LI15’’’’’f); when mature bowl-shaped to discoid, near point of attachment Slate-Gray (LIIICARBON GRAYi) to Deep Neutral Gray (LIIINEUTRAL GRAYi), center Gray (Deep Gull Gray) (LIIICARBON GRAYb) to Neutral Gray (LIIINEUTRAL GRAY), margin Light Neutral Gray (LIIINEUTRAL GRAYb) to Gray (Deep Gull Gray) (LIIICARBON GRAYb); surface covered with pruina, White (LIIINEUTRAL GRAY) to Gray (Pale Gull Gray) (LIIICARBON GRAY), base with coarse felt, Gray (Light Gull Gray) (LIIICARBON GRAYf) to Gray (Dark Gull Gray) (LIIICARBON GRAY); faintly striate, not hygrophanous. ***Context*** 1 mm thick, translucent, gelatinous, tough, elastic, not fragile. ***Hymenophore*** lamellate; ***lamellae*** thin, < 1 mm broad, *L* = 7–11, *l* = 3–11, free, Pale Olive-Gray (LI15’’’’’f) to Light Olive-Gray (LI23’’’’’b) when young, Light Olive-Gray (LI23’’’’’b) to Mouse Gray (LI69’’’’f) when mature, surface pruinose, gelatinous. ***Stipe*** absent, basidiomata laterally or dorsally attached. Odour and taste indistinct.

***Basidiospores*** (200/8/4) 4.5–***4.6***–4.7 × 4.3–***4.4***–4.5 μm, [Q = 1.04–1.08, Qm = ***1.07*** ± 0.01] [holotype (50/2/1) 4.6–***4.6***–4.7 × 4.3–***4.4***–4.5 μm, Q = 1.06–1.07, Qm = ***1.07*** ± 0.01], globose to subglobose, smooth, thin-walled, hyaline, with circular or irregular guttules, inamyloid. ***Basidia*** 12–21 × 4–7 μm, cylindrical to clavate, 2- or 4-spored, sterigmata 2.7–4.4 μm long, base constricted, thin-walled, smooth, hyaline, with guttules. ***Cheilocystidia*** coralloid to cervicorn, 9–19 × 2–7 μm, mostly branched, with apical digitate protrusions 2.0–4.4 μm long, thin-walled, hyaline. ***Pleurocystidia*** absent. ***Pileipellis*** a trichocutis, with a gelatinous layer, non-dextrinoid, composed of cylindrical hyphae 2–3 μm broad, thin-walled, hyaline, occasionally with spinose protrusions 2.3–3.5 μm long; terminal hyphae slightly inflated, branched, branches developing into erect, smooth, thick-walled hyphae 2–3 μm broad, containing dark brown intracellular pigment; these hyphae interwoven in tufts, forming a trichoderm; crystals occasionally present. ***Lamellar trama*** subregular, with a gelatinous layer, non-dextrinoid, composed of subparallel, cylindrical hyphae 2–4 μm broad, occasionally with spinose protrusions 2.4–4.3 μm long, thin-walled, hyaline, with conspicuous crystals. Clamp connections present in all tissues.

##### Habit and habitat.

Scattered on well-decayed wood and dead branches in temperate to subtropical mixed coniferous and broad-leaved forests, often dominated by *Fagaceae*, *Pinaceae*, and *Salicaceae*.

##### Known distribution.

Shandong Province and Zhejiang Province, China.

##### Additional specimens examined.

China • Shandong Province, Linyi City, Mengyin County, Mengshan National Forest Park, 20 July 2021, Guanyu Qiu, Yupeng Ge, Qin Na, Hui Zeng, Zewei Liu, Yulan Sun, and Menghui Han, 800 m alt., *FFAAS3504* (collection no. GN1182); • Shandong Province, Linyi City, Mengyin County, China Waterfall, Mengshan, 21 July 2021, Guanyu Qiu, Yupeng Ge, Qin Na, Hui Zeng, Zewei Liu, Yulan Sun, and Menghui Han, 900 m alt., *FFAAS3505* (collection no. GN1217); • Zhejiang Province, Hangzhou City, Tianmu Mountain Scenic Area, 30 July 2021, Guanyu Qiu, Yupeng Ge, Qin Na, and Zewei Liu, 1,506 m alt., *FFAAS3506* (collection no. GN1327).

##### Notes.

*Resupinatus cinereofuscus* resembles *R. applicatus*, *R. hausknechtii* Consiglio & Setti, and *R. rouxii* Consiglio & Setti in having thin lamellae, coarse felt at the point of attachment, and globose to subglobose basidiospores (Roux 2006; McDonald 2015; Consiglio and Setti 2018). However, *R. applicatus* and *R. hausknechtii* are characterized by spatulate to flabelliform basidiomata and non-striate pilei (McDonald 2015; Consiglio and Setti 2018). Furthermore, *R. applicatus* has cylindrical, branched, twisted pleurocystidia, and its pileipellis hyphae have distinct branching, coral-like or brown, coarse, wart-like protrusions, whereas the basidiomata of *R. hausknechtii* are white (McDonald 2015; Consiglio and Setti 2018). *Resupinatus rouxii* is characterized by a larger, dark brown pileus (1.5–2.5 cm in diam.) covered with gray tomentum and pileipellis hyphae containing both intracellular and encrusting pigment (Roux 2006; Consiglio and Setti 2018). In addition, *R. cinereofuscus* could be confused with *R. cinerascens*, but the latter is distinguished by cupulate to convex basidiomata, a pileus up to 15 mm in diam., ovoid to ellipsoid basidiospores, and basidia turning brown in KOH (McDonald 2015).

### Key to the 49 species of *Resupinatus*

**Table:** 

1	Hymenophore lamellate	**2**
–	Hymenophore not lamellate	**30**
2	Lamellae branched	**3**
–	Lamellae unbranched	**8**
3	Pileus covered with tomentose	**4**
–	Pileus without tomentum	**7**
4	Pileus sulcate	***R. chilensis***
–	Pileus not sulcate	**5**
5	Basidia turn brown in KOH	***R. cinerascens***
–	Basidia don’t turn brown in KOH	**6**
6	Pileipellis hyphae with asterostromelloid elements	***R. vinosolividus***
–	Pileipellis hyphae without asterostromelloid elements	***R. meihui***
7	Pileus striate	***R. odoratus***
–	Pileus not striate	***R. merulioides***
8	Central or lateral stipe present	***R. omphalioides***
–	Stipe absent	**9**
9	Pileus smooth	***R. porrigens***
–	Pileus not smooth	**10**
10	Pileus covered with tomentose	**11**
–	Pileus without tomentum	**15**
11	Lamellae white	**12**
–	Lamellae not white	**13**
12	Pileus campanulate	***R. campanulatus***
–	Pileus not campanulate	***R. sordulentus***
13	Pileus striate	**14**
–	Pileus not striate	***R. algidus***
14	Pileus cupulate to discoid	***R. subrhacodium***
–	Pileus lenticular to subcircular or reniform	***R. atropellitus***
15	Pileus pruinose	**16**
–	Pileus not pruinose	**25**
16	Pileus striate	**17**
–	Pileus not striate	**19**
17	The point of attachment covered with coarse felt	***R. cinereofuscus***
–	The point of attachment without coarse felt	**18**
18	Basidiospores globose to subglobose, Q = 1.04–1.21	***R. striatulus***
–	Basidiospores cylindrical to allantoid, Q = 2.01–2.64	***R. alboniger***
19	The point of attachment covered with coarse felt	**20**
–	The point of attachment without coarse felt	**22**
20	Cheilometuloids present	***R. niger***
–	Cheilometuloids absent	**21**
21	Basidiospores globose to subglobose, Q = 1.02–1.17	***R. applicatus***
–	Basidiospores globose, Q = 1.05–1.07	***R. sinoapplicatus***
22	Lamellae absent or imperfectly formed	***R. kavinae***
–	Lamellae perfectly formed	**23**
23	The point of attachment covered with mycelial fibrils	***R. europaeus***
–	The point of attachment without mycelial fibrils	**24**
24	Pileipillis hyphae with wavy, diverticulate, and utriform protrusions	***R. abieticola***
–	Pileipillis hyphae without protrusions	***R. americanus***
25	Basidiomata white	***R. hausknechtii***
–	Basidiomata not white	**26**
26	Pileus striate	**27**
–	Pileus not striate	**28**
27	Lateral stipe present	***R. dealbatus***
–	Stipe absent	***R. reviviscens***
28	Pleurocystidia present	***R. trichotis***
–	Pleurocystidia absent	**29**
29	Pileipellis hyphae coralloid-branched	***R. subapplicatus***
–	Pileipellis hyphae not coralloid-branched	***R. rouxii***
30	Hymenophore poroid	**31**
–	Hymenophore cyphelloid	**34**
31	Pileus striate	***R. porosus***
–	Pileus not striate	**32**
32	Pores circular to subcircular	***R. latemarginatus***
–	Pores not circular	**33**
33	Basidiospores curved, cylindrical to allantoid	***R. angulatus***
–	Basidiospores ellipsoid	***R. sinuosus***
34	Basidiomata white	**35**
–	Basidiomata not white	**36**
35	Context hyaline and translucent	***R. hyalinus***
–	Context white	***R. taxi***
36	Basidiomata seated in subiculum	**37**
–	Basidiomata not seated in subiculum	**46**
37	Subiculum white	**38**
–	Subiculum not white	**42**
38	Basidiomata black	**39**
–	Basidiomata not black	**41**
39	Pileus margin incurved	**40**
–	Pileus margin straight	***R. huia***
40	Tomentose hyphae of the pileus covered with crystals	***R. cinereus***
–	Tomentose hyphae of the pileus without crystals	***R. daedalea***
41	Basidiospores globose to subglobose	***R. poriaeformis***
–	Basidiospores cylindrical	***R. incanus***
42	Subiculum felt-like	**43**
–	Subiculum not felt-like	**44**
43	Cystidioles present	***R. tenuis***
–	Cystidioles absent	***R. yunnanensis***
44	Subiculum covered with crystals	**45**
–	Subiculum without crystals	***R. conspersus***
45	Basidiospores globose to subglobose	***R. urceoloides***
–	Basidiospores cylindrical	***R. conglobatus***
46	Pileus pruinose	**47**
–	Pileus not pruinose	**48**
47	Hymenophore grayish black to black	***R. urceolatus***
–	Hymenophore hyaline and translucent	***R. rickii***
48	Cystidia present	***R. stictoideus***
–	Cystidia absent	***R. griseopallidus***

## Discussion

Relying on the surface covering of the pileus is often insufficient to distinguish species within *Resupinatus*, frequently leading to taxonomic confusion (Berkeley 1873; Pilát 1935; Largent et al. 1977; Largent and David 1986; Bas et al. 1995; Ludwig 2000). In contrast, basidiospore morphology, as well as the presence and morphology of cystidia, exhibits clear differences among related species (Berkeley 1873; Pilát 1935; Largent et al. 1977; Largent and David 1986; Bas et al. 1995; Ludwig 2000). For example, *R. applicatus* and *R. trichotis* share similar cupulate to flabelliform basidiomata, gray to blackish-gray pileus, and lamellate hymenophore (Pilát 1935; Bas et al. 1995; Ludwig 2000; Consiglio and Setti 2018). There are differences in the color of the mycelial felt attached to the pileus base, but these differences can be observed only in basidiomata that have not aged and in which the mycelial felt is still observable (Consiglio and Setti 2018). In contrast, the two species exhibit distinct differences in the morphology of their basidiospores and cystidia. *R. applicatus* has subglobose to slightly lacrymoid basidiospores and shorter, clavate cheilocystidia with coarse and fine finger-like projections, whereas *R. trichotis* has globose basidiospores and longer, clavate to ventricose cheilocystidia with tortuous, finger-like projections (Thorn and Barron 1986; Consiglio and Setti 2018). *Resupinatus meihui* has cylindrical to clavate cheilocystidia, whereas the related *R. vinosolividus* has sinuato-cylindrical to ventricose cheilocystidia. Furthermore, *R. cinereofuscus* has globose to subglobose basidiospores, whereas the related *R. applicatus* has subglobose to slightly lacrymoid basidiospores (McDonald 2015; Consiglio and Setti 2018). In the phylogenetic tree, *R. meihui* and *R. vinosolividus* clustered in the same clade with strong support (BS/BPP = 100/1.00), but each formed an independent lineage with high support (BS/BPP = 100/0.99 and 100/0.96, respectively). Specimens in the *R. meihui* lineage are characterized by broadly ellipsoid to subglobose basidiospores, Q = (1.08)1.13–1.35, which are distinct from the ellipsoid to elongate-ellipsoid basidiospores of *R. vinosolividus*, Q = 1.6. Similarly, *R. cinereofuscus* and *R. applicatus* were resolved as sister taxa with strong support (BS/BPP = 100/1.00), each forming an independent lineage with high support (BS/BPP = 100/0.99). The *R. cinereofuscus* lineage lacked pleurocystidia, distinguishing it from the *R. applicatus* lineage.

Studies from China and other regions indicate that species of *Resupinatus* differ markedly in substrate types and elevational ranges, whereas their distributions across climatic zones are relatively consistent (Singer 1986; Thorn et al. 2000, 2005; Bau et al. 2007, 2013; Cooper 2012; Li and Bau 2014; McDonald 2015; Consiglio and Setti 2018). In terms of substrate, *Resupinatus* species reported outside China mainly occur on the dead branches and rotten wood of both coniferous and broad-leaved trees, including *Pinus*, *Abies*, *Cupressus*, *Taxus*, *Quercus*, *Fagus*, *Acer*, *Betula*, *Populus*, *Salix*, *Ulmus*, *Fraxinus*, and *Eucalyptus*. In addition, some species occur less frequently on dead branches or decaying leaves of grapevines, bamboo, and palms (Singer 1986; Redhead and Nagasawa 1987; Segedin 1993; Thorn et al. 2000, 2005; Martín et al. 2006; Gonou-Zagou et al. 2011; Cooper 2012; McDonald 2015; Consiglio and Setti 2018; Bijeesh et al. 2020; Akçay 2021; Carpouron et al. 2024; Vizzini et al. 2024; Triantafyllou et al. 2026). In China, species of *Resupinatus* are commonly found on dead branches and rotten wood of coniferous and broad-leaved trees, such as *Juniperus*, *Picea*, *Tamarix*, *Nitraria*, *Platycladus*, *Cinnamomum*, *Camellia*, *Betula*, and *Rhododendron* (Teng 1963; Huang 1998; Lu 2000; Bau et al. 2007, 2013; Wu et al. 2009, 2011; Li and Bau 2014; Yang et al. 2023; Liu et al. 2024; Dong et al. 2025; Zhuang et al. 2026). With respect to elevation, more than half of the known *Resupinatus* species outside China occur at low elevations below 1,000 m, approximately one-third occur at middle elevations of 1,000–3,500 m, and no species have been recorded at high elevations above 3,500 m (Singer 1986; Thorn et al. 2000, 2005; McDonald 2015; Consiglio and Setti 2018). By contrast, species of *Resupinatus* reported from China are known mostly from middle elevations of 1,000–3,500 m, whereas very few occur at low elevations below 1,000 m (Teng 1963; Huang 1998; Lu 2000; Wu et al. 2009, 2011; Li and Bau 2014; Yang et al. 2023; Liu et al. 2024; Dong et al. 2025; Zhuang et al. 2026). In terms of climatic zones, species of *Resupinatus* reported from China and other regions show a similar distribution pattern. They are mainly reported from subtropical regions, followed by temperate and tropical regions, and no species have been recorded from the Arctic zone (Singer 1986; Thorn et al. 2000, 2005; Li and Bau 2014; McDonald 2015; Consiglio and Setti 2018; Yang et al. 2023; Carpouron et al. 2024; Liu et al. 2024; Vizzini et al. 2024; Dong et al. 2025; Triantafyllou et al. 2026; Zhuang et al. 2026).

## Supplementary Material

XML Treatment for Resupinatus meihui

XML Treatment for Resupinatus cinereofuscus

## Additional information

### Artificial Intelligence (AI) use

The authors accept full responsibility for the content of the manuscript, including the disclosure of any use of AI.

No AI tools were used in the preparation of this manuscript.

### Author ORCIDs

G. Qiu https://orcid.org/0009-0000-2349-2296

Y. Ge https://orcid.org/0000-0001-5754-201X

H. Zeng https://orcid.org/0000-0003-2025-844X

T. Wang https://orcid.org/0009-0004-0573-9530

Q. Na https://orcid.org/0000-0001-8406-6389

Y. Hu https://orcid.org/0000-0003-1242-1139

### Data availability

All of the data that support the findings of this study are available in the main text or Supplementary Information.

## Supplementary materials

Supplementary material 1 Fasta of the phylogenetic tree Data type fas https://binary.pensoft.net/file/1778184This dataset is made available under the Open Database License (http://opendatacommons.org/licenses/odbl/1.0/). The Open Database License (ODbL) is a license agreement intended to allow users to freely share, modify, and use this Dataset while maintaining this same freedom for others, provided that the original source and author(s) are credited. Guanyu Qiu, Yupeng Ge, Hui Zeng, Tian Wang, Qin Na, Yaping Hu
